# Activity in an air–liquid interface lung infection model, feasibility of inhaled delivery, and stability of cell-free supernatants from *Lacticaseibacillus rhamnosus* against *Pseudomonas aeruginosa* pulmonary infections

**DOI:** 10.3389/fmicb.2025.1630017

**Published:** 2025-08-05

**Authors:** Anna Maria Piras, Marta Bianchi, Nicolò Della Bona, Brunella Grassiri, Esingül Kaya, Andrea Bertacca, Chiara Migone, Giuseppantonio Maisetta, Semih Esin, Giovanna Batoni

**Affiliations:** ^1^Department of Pharmacy, University of Pisa, Pisa, Italy; ^2^Centre for Instrument Sharing of the University of Pisa (CISUP), Pisa, Italy; ^3^Department of Translational Research and New Technologies in Medicine and Surgery, University of Pisa, Pisa, Italy

**Keywords:** *Lacticaseibacillus rhamnosus*, *Pseudomonas aeruginosa*, lung infections, cystic fibrosis, solution for inhalation, lung model

## Abstract

**Objective:**

Given the increasing prevalence of multidrug-resistant pathogens and the diminishing efficacy of conventional antibiotics, this study explores the potential of probiotics or their metabolic products as alternative antimicrobial agents. Specifically, we investigated the antibacterial properties of cell-free supernatants (CFS) derived from the probiotic strain *Lacticaseibacillus rhamnosus* GG for the local treatment of *Pseudomonas aeruginosa* lung infections.

**Methods:**

To simulate the human respiratory environment, we employed various *in vitro* models. The cytotoxicity and antibacterial activity of CFS were assessed using an Air-Liquid Interface (ALI) lung infection model based on differentiated NCI-H441 human distal lung epithelial cells cultured on Transwell® inserts. To evaluate the feasibility of aerosol-based delivery, we developed and characterized a liquid formulation of CFS. The aerodynamic performance of nebulized CFS was analyzed using a twin-stage impinger (TSI) and a Next Generation Impactor (NGI), the latter equipped with a breathing simulator to mimic respiratory profiles of both healthy individuals and cystic fibrosis patients. Additionally, the physicochemical and biological stability of CFS was assessed under various storage conditions.

**Results:**

CFS demonstrated significant antibacterial activity in the ALI model, reducing *P. aeruginosa* colony-forming units by up to 3 log units after 7 h of incubation, without inducing cytotoxic effects. Scanning electron microscopy confirmed these findings. Aerodynamic testing with the TSI and an Aerogen® mesh nebulizer showed that 76% of the nebulized product was deposited in the second stage, indicating effective deep lung delivery. NGI analysis revealed a favorable aerodynamic particle size distribution (APSD), with a fine particle fraction (FPF) exceeding 60% and a mass median aerodynamic diameter (MMAD) suitable for deep airway deposition. Physicochemical stability studies under stressed temperature conditions predicted prolonged physical stability for CFS at 25°C and demonstrated that they retained anti-pseudomonal activity after 1 year of storage at room temperature, 4°C, and −20°C.

**Conclusion:**

These findings support the potential of *L. rhamnosus* GG-derived CFS as a promising candidate for inhaled therapy against *P. aeruginosa* lung infections. Further validation in animal models is warranted to confirm its therapeutic efficacy and safety *in vivo*, potentially contributing to the development of novel localized treatment strategies for respiratory infections.

## 1 Introduction

Antimicrobial resistance (AMR) represents a growing threat to human health all around the world. A recent systematic analysis reported an estimate of almost 5 million deaths associated with bacterial AMR in 2019, of which 1.27 million were directly attributable to bacterial AMR (Murray et al., 2022). The current antibiotic development pipeline does not adequately address the emergence of multi-, extensively-, and pan-resistant bacterial pathogens. As a result, the development of innovative antimicrobial strategies is widely recognized as a research priority (Ajose et al., 2024; Piddock et al., 2024; Ralhan et al., 2024).

*Pseudomonas aeruginosa* is an opportunistic Gram-negative bacterium currently representing one of the major threats in clinical settings due to its ability to express a variety of virulence factors, form biofilms, and infect disparate body districts. In addition to its multifaceted pathogenicity, the bacterium expresses multiple resistant mechanisms even toward last-resort antibiotics, rendering problematic its eradication with the current arsenal of antimicrobial drugs (Horcajada et al., 2019; Kothari et al., 2023). In hospital settings, *P. aeruginosa* is often involved in highly deadly acute pneumonia (Barp et al., 2023), but it can also cause chronic lung infections, especially in patients with underlying diseases such as cystic fibrosis (CF), non-CF bronchiectasis (NCFB), and chronic obstructive pulmonary disease (COPD) (Martínez-Solano et al., 2008; Wilson et al., 2016; Alqasmi, 2024).

The challenges in treating multidrug *P. aeruginosa* infections have stimulated the search for antibiotic alternatives to target this bacterium, including quorum sensing inhibitors (Rodríguez-Urretavizcaya et al., 2024), phages (Martinho et al., 2024), antimicrobial peptides (Maisetta et al., 2017; Grassi et al., 2019), and others. Among these alternatives, lung delivery of probiotics has recently gained an intense interest as it could correct the dysbiotic microbiota at the pulmonary level while controlling the growth and virulence of respiratory pathogens (Batoni et al., 2022; Byun et al., 2023; Glieca et al., 2024b). As proof of concept, Fangous and coworkers demonstrated that intra-tracheal administration of lactobacilli protects mice from *P. aeruginosa* pulmonary infection (Fangous et al., 2019). Likewise, intranasal delivery of probiotics was shown to synergistically interact with an anti-infective antibody and improve mice survival after primary and secondary infection with *P. aeruginosa* while exerting an immune-modulating effect (Sécher et al., 2024).

Inhalation powders containing lactic acid probiotics (i.e., *Lactiplantibacillus plantarum*, *Lacticaseibacillus rhamnosus*, or *Lactobacillus acidophilus*) have been recently developed in order to assess the feasibility of their administration to the lungs of patients with *P. aeruginosa* respiratory infections (Glieca et al., 2024a). The feasibility of nebulization as a modality to deliver *L. rhamnosus* to the lung has also been recently investigated, demonstrating that the efficiency of the process depends on the nebulization type and the liquid medium of the probiotic suspension (Byun et al., 2023). Interestingly, we have previously reported that in an *in vitro* system, *L. plantarum* and *L. rhamnosus* exert strong antibacterial and antibiofilm properties against lung isolates of *P. aeruginosa* (Batoni et al., 2023a), while *L. acidophilus* effectively prevents adhesion of the same isolates to lung epithelial cells (Batoni et al., 2023b), suggesting possible mechanisms of probiotic-dependent *P. aeruginosa* control at the lung level.

Despite these promising findings, current research supports the view that the beneficial health effects of probiotics are only partially ascribed to their vitality, as probiotic-derived inanimate preparations (e.g., non-viable microbes, their cell components, or their metabolites) can also exert biological activities with therapeutic potential (Aggarwal et al., 2022; da Silva Vale et al., 2023). These findings have led to the evolving concept of postbiotics, originally defined as metabolic products of probiotics, although this definition has recently been revised (Aguilar-Toalá et al., 2021; Vinderola et al., 2022). As postbiotics may overcome some of the safety concerns related to the use of live probiotics (e.g., transmission of antibiotic resistance, possibility of systemic infections in vulnerable individuals, challenges in maintaining vitality during processing and storage), their preclinical characterization as anti-infective agents is worth investigating.

We previously reported that postbiotics from *L. rhamnosus* in the form of cell-free supernatants (CFS) exert strong antibacterial, antibiofilm, and antivirulence properties against clinical isolates of *P. aeruginosa* (Pompilio et al., 2024). Importantly, we have also demonstrated that they act even against antibiotic-refractory persister cells (Bianchi et al., 2024) and do not induce resistance following multiple passages at sub-inhibitory concentrations, unlike what was observed with a conventional antibiotic (Kaya et al., 2024).

In this study, we sought to investigate further, at a preclinical level, the translational potential of CFS for the local therapy of lung infections by *P. aeruginosa* as an alternative to live bacteria. To this aim, we tested the antipseudomonal activity of CFS in an Air-Liquid interface (ALI) lung infection model in order to mimic *in vitro* the conditions found in the infected airways. In the same model, the cytotoxic potential toward a human lung epithelial cell line was also explored. Furthermore, the use of *L. rhamnosus* postbiotics as a solution for nebulization was investigated, evaluating its aerodynamic distribution under normal adult and CF patients’ breathing profiles. Finally, physicochemical stability studies were performed under stressed temperature conditions, and the antimicrobial activity maintenance of postbiotics under different storage conditions was investigated.

Overall, our findings demonstrate that CFS exhibit effective anti-bacterial activity at non-toxic concentrations in the ALI lung infection model against clinical isolates of *P. aeruginosa* and the potential to reach the deeper regions of the lung. In addition, they exhibit high stability in a number of storage conditions. These results form the basis for innovative strategies for the local treatment of *P. aeruginosa* lung infections.

## 2 Materials and methods

### 2.1 Bacterial strains used in the study

Two clinical strains of *P. aeruginosa* from our collection were used in the study, named PA1 and PA4. Both of them were isolated from the *sputum* of patients with CF, chronically infected with the bacterium and exhibited a non-mucoid (PA1) and a mucoid (PA4) phenotype, respectively. The strains were identified by using a MALDI-TOF Microflex LT Mass Spectrometer (MALDI-TOF-MS) equipped with a MALDI Biotyper 3.1 software (Bruker Daltonics; Bremen, Germany) according to the manufacturer’s instructions. The Phoenix System (Becton Dickinson Italia; Milan, Italy) was used for antibiotic susceptibility testing. The antibiotic susceptibility profile of the isolates has been previously reported (Pompilio et al., 2024).

### 2.2 Preparation of CFS

*L. rhamnosus* was isolated from a dietary supplement purchased from a local pharmacy (Microbiosys, Sanofi, Vitry-Sur-Seine, France) and identified at the species level by MALDI-TOF-MS as previously described (Pompilio et al., 2024). Strain identification was carried out by Novogene (Beijing, China) through whole genome sequencing and data analysis, revealing a mapping rate of 99.83% with *L. rhamnosus* strain GG’s reference genome. The strain was grown overnight in De Man-Rogosa-Sharpe broth (MRSB, Oxoid, Basingstoke, Hampshire, UK). The bacterial suspension was then diluted 1:100 in fresh MRSB and incubated at 37°C for 48 h in shaking conditions. Following incubation, the culture was centrifuged at 4,000 × g for 10 min, and supernatants were collected and filtered through 0.22 μm filters. CFS were divided into aliquots kept at −20°C until use.

### 2.3 ALI lung infection model

The human distal lung epithelial cell line NCI-H441 (ATCC HTB-174) was routinely grown in RPMI 1640 medium (Euroclone, Pero, Milan, Italy) supplemented with 2 mM L-glutamine (Euroclone, Pero, Milan, Italy) and 10% fetal bovine serum (Euroclone, Pero, Milan, Italy) (complete medium) at 37°C in humidified atmosphere containing 5.5% CO_2_. At the time of the experiment, cells were seeded onto 0.33 cm^2^ tissue culture inserts with 0.4 μm diameter pores (Sarstedt, Numbrecht, Germany) at a density of 2.5 × 10^4^ cells/well and incubated in complete medium for 48 h. Following incubation, the medium from the upper chamber was removed, leaving the apical surface of the cells exposed to air, while that of the lower chamber was removed and replaced with complete medium containing 1% insulin-transferrin-selenium (ITS) and 200 nM dexamethasone (Merck, Darmstadt, Germany). The medium was changed every 2–3 days. Following 7–8 days of incubation, transepithelial electrical resistance (TEER) was measured (Voltohmmeter millicell-ERS 2.0, Merck, Darmstadt, Germany) to evaluate whether the differentiation had occurred. A value of approximately 600–700 Ω/cm^2^, was considered indicative of a continuous differentiated monolayer. The monolayers were then infected with *P. aeruginosa* PA1 or PA4 at a multiplicity of infection (MOI) of 1:25 bacteria:cell. In a first set of experiments, *P. aeruginosa* growth in the model was monitored at 0, 7, 17, and 25 h. At each time-point, a 200X light microscope photo of the infected cells was taken, and bacteria were collected from the wells by three consecutive washes with 0.1% TritonX-100 (Merck, Darmstadt, Germany) in water. The 3 washes from each well were unified, vigorously vortexed, serially diluted in PBS, and plated on Tryptic Soy Agar (TSA, Oxoid, Basingstoke, Hampshire, UK). Colony-forming unit (CFU) enumeration was performed after 24 h incubation of the plates at 37°C. For the treatment experiments, CFS diluted 1:4 or 1:8 in complete medium was added to wells simultaneously with *P. aeruginosa* and kept for 7 h. Untreated controls, incubated with 1:4 or 1:8 MRSB in complete medium, were established in parallel. At the end of the incubation period, bacterial load in treated samples and untreated controls was determined as explained above.

### 2.4 Scanning electron microscopy images of *Pseudomonas aeruginosa*-infected monolayers

Imaging of TC-inserts containing: (A) differentiated NCI-H441 cells alone; (B) differentiated NCI-H441 cells infected with *P. aeruginosa* and left untreated; (C) differentiated NCI-H441 cells infected with *P. aeruginosa* and treated with CFS 1:8, was performed by scanning electron microscopy (SEM). To this aim, wells were washed 3 times with PBS to remove non-attached bacteria and fixed with 2.5% glutaraldehyde (Merck, Darmstadt, Germany) at 4°C for 2.5 h. The specimens were then subjected to increasing concentrations of ethanol (Merck, Darmstadt, Germany) (25, 50, 75, 95, and 100%) for dehydration. The dehydrated specimens were dried under a flow laminar hood overnight and examined by field emission SEM FEI Quanta 450 FEG from the Center for Instrument Sharing of the University of Pisa, Italy (CISUP). Images were acquired from randomly selected areas on each sample using 6,000×, 12,000×, and 24,000 × magnifications.

### 2.5 Cytotoxicity assay

The cytotoxic potential of the CFS was tested against differentiated NCI-H441 cells in the *in vitro* ALI model. To this aim, cells in complete RPMI were exposed to CFS at 1:4 and 1:8 dilutions for 7 h at 37°C in a humidified atmosphere containing 5.5% CO_2_. The same medium was added to the inferior chamber of the wells. Negative controls consisted of cells in complete RPMI exposed to MRSB diluted 1:4 in complete RPMI, and positive controls consisted of cells exposed to 0.1% Triton-X 100. Following incubation, the cells were washed once with 200 μL of warm PBS and subjected to trypsin/ethylenediaminetetraacetic acid (EDTA, Euroclone, Pero, Milan, Italy) treatment at 37°C for 3 min. Following a wash with PBS at 700 × g the pellets were resuspended in PBS. Cells were stained for 5 min with 0.1 μg/mL propidium iodide (Merck, Darmstadt, Germany) at room temperature before performing flow cytometric acquisition. To this aim, 50,000 events were acquired ungated by using a flow cytometer (BD Accuri C6, BD Biosciences, Italy). The percentage of PI-positive NCI-H441 cells was calculated by computer-assisted analyses (BD Accuri C6 software version 1.0.264.21, BD Biosciences, Milan, Italy) and the percent of vitality was calculated according to the following formula: 100 − [100 × (%PI pos cells sample − %PI pos cells neg control)]/(100 − % PI pos cells neg control).

### 2.6 CFS as solution for nebulization

The pH of the CFS was evaluated with the Bench Meter pH 50 VioLAb Bioclass XS (Mettler-Toledo S.p. A., Milan, Italy). The osmolarity of the supernatants was also determined (Osmomat 3000, Gonotec Berlin, Germany). Lactic acid quantification was performed by ultra-high-performance liquid chromatography (UHPLC) coupled to a high-resolution electrospray ionization source Orbitrap-based mass spectrometer (HR-ESI-Orbitrap/MS). The LC–MS system was composed of a Vanquish Flex Binary pump LC and an ESI Q Exactive Plus MS, Orbitrap-based FT-MS system (Thermo Fischer Scientific Inc., Darmstadt, Germany). The samples were diluted in methanol (1:10), and 5 μL each were injected on a C-18 Kinetex® Biphenyl column (100 × 2.1 mm, 2.6 μm particle size) provided by a Security GuardTM Ultra Cartridge (Phenomenex, Bologna, Italy). The elution was performed at a flow rate of 0.3 mL/min with 0.1% formic acid in H_2_O (solvent A) and 0.1% formic acid in methanol (solvent A) by using the following solvent gradient: 0–2.0 min, isocratic 20% B; 2.0–3.0 min, 20–30% B; 3.0–5.0 min, 30–95% B; 5–0.0–6.0 min, isocratic 95% B; 6.0–7.0 min, 95-20%B. The temperature of the autosampler and column oven was maintained at 4°C and 35°C, respectively. HR mass spectra were acquired in ESI negative ionization modes (scan range m/z 50–500) operating in full (70,000 resolution, 220 ms maximum injection time). The lactic acid calibration curve was established in the range of 1–50 mg/mL (R^2^:0.997); CFS samples were diluted 1:100 before analysis. Lactic acid was also determined by L-Lactate Assay Kit MAK329 (Merck, Darmstadt, Germany).

### 2.7 Aerodynamic particle size distribution and delivery dose uniformity assessment

Preliminary aerodynamic evaluation of the aerosol clouds was performed by using a glass twin-stage impinger (TSI), according to European Pharmacopoeia 11.1 ed., 2.9.18 Apparatus A procedure (European Pharmacopoeia, 2023) and as previously reported in (Vizzoni et al., 2023). CFS was loaded in an Aerogen® Solo mesh nebulizer (Aerogen Ltd., Galway, Ireland), and 2 mL of sample were nebulized (5 min ± 30 s). Afterwards, the upper and lower-stage solutions were collected individually, and lactic acid content was quantified by LC–MS. The procedure was repeated three times. The results for each of the two parts of TSI were expressed as a percentage of the total amount of active substance.

The delivery dose uniformity (DDU) and aerodynamic particle size distribution (APSD) were evaluated using a Next Generation Impactor (NGI, Copley, UK) equipped with a breathing simulator (BRS300i, Copley, UK). Two different breathing profiles were applied: healthy adult and adult CF profiles. The healthy adult breathing profile had a tidal volume of 500 mL, a rate of 15 breaths per minute (BPM), an inspiratory-expiratory (I: E) ratio of 1:1, and a breathing cycle of 4 s. The CF adult profile had a tidal volume of 525 mL, a rate of 22 BPM, an I: E ratio of 1:4, and a breathing cycle of 2.7 s (Tiddens et al., 2000; Sweeney et al., 2015). A total of 2 mL of CFS was nebulised over a period of 5 min using the handheld device Aerogen® Ultra mesh nebulizer, operating at a flow rate of approximately 0.45 mL/min. Initially, DDU was determined on propylene filters (low resistance filter, PARI, Germany) contained within a filter holder interfaced with the device through a mouthpiece adapter (Council of Europe: Strasbourg, France, 2014). Subsequently, APSD was assessed using the NGI, which was placed in a cooler at 5°C for 90 min prior to the experiment. Lactic acid was used as a deposition tracer, and its concentration was quantified using the L-Lactate Assay Kit MAK329. The procedures were repeated five times.

### 2.8 Physicochemical CFS stability under stress temperature conditions

Physicochemical stability studies were performed under stress temperature conditions (40–90°C) for 1 week with CFS stored in sealed vials. Sample-forced degradation was monitored via UV absorbance, and degradation rates were calculated and applied to the Arrhenius equation to predict the stability of the solution at both room temperature and refrigerated conditions. A Lambda 25 UV/VIS Spectrometer (Perkin Elmer, Milan, IT) was used for the analysis, with samples diluted at a 1:20 ratio. A complete UV spectrum was recorded for each sample across a wavelength range of 200–800 nm; the absorbance at 450 nm was used for degradation monitoring.

### 2.9 Evaluation of the antibacterial activity of CFS exposed to stress temperature conditions

Representative CFS samples, namely those treated at 60°C, 80°C, or 90°C for 24 h, and at 90°C for 5 or 7 days, were tested for antibacterial activity against *P. aeruginosa* PA1 by performing killing assays. Planktonic PA1 cells in exponential phase (1 × 10^7^ CFU/mL) were incubated for 45 min at 37°C with stressed CFS diluted 1:4 in TSB, in a total volume of 500 μL. Controls consisted in bacteria exposed to (a) MRSB diluted 1:4 in TSB; (b) the same CFS batch diluted 1:4 in TSB not subjected to stress temperature conditions. Following incubation, samples were serially diluted in PBS and plated on TSA for CFU enumeration.

### 2.10 Evaluation of the antibacterial activity of CFS in different storage conditions

The stability of CFS was evaluated following 12 months of storage under different conditions. To this aim, freshly prepared CFS were aliquoted and stored at 25°C, 4°C, and −20°C. After 1 year, aliquots from each storage condition were tested for antibacterial activity against *P. aeruginosa* PA1 by a broth microdilution assay in 96-well plates (Euroclone, Pero, Milan, Italy). In the same assay, fresh CFS samples were included as a control for antibacterial activity. All the CFS samples were used at the final dilutions of 1:4, 1:8, 1:16, 1:32, and 1:64. Bacteria resuspended in TSB from an overnight culture were added to each well to reach a final concentration of 5 × 10^5^ CFU/mL. Controls consisted of bacteria incubated in TSB/MRSB and TSB/MRSB without bacteria. Plates were sealed with a breathable film and incubated at 37°C in an ELISA reader with OD_620_ measurements taken every 30 min for 24 h, with shaking prior to each reading. All OD values were normalized by subtracting the corresponding time-zero measurement.

### 2.11 Statistical analysis

Each experiment was performed at least in triplicate. The statistical analysis was performed using GraphPad Prism 9.0 software (GraphPad Software, San Diego, California, United States). One or Two-way ANOVA, followed by Tukey–Kramer’s multiple comparisons test was applied when assessing differences among 3 or more groups of unpaired data. A *p*-value < 0.05 was considered statistically significant.

## 3 Results

### 3.1 *Pseudomonas aeruginosa* growth in the ALI model

**Figure 1:**
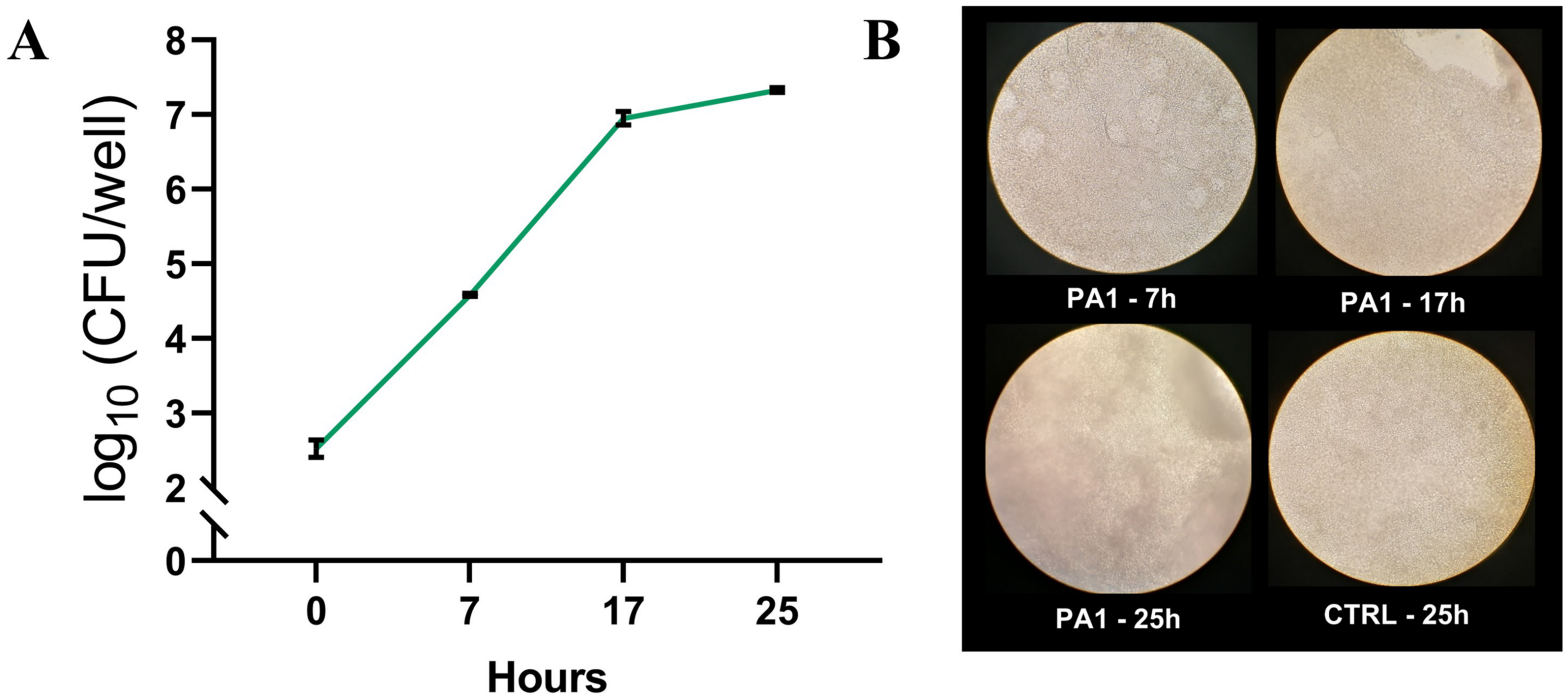
*P. aeruginosa* growth in the ALI model. PA1 was used to infect differentiated lung epithelial NCI-H441 cells at a multiplicity of infection of 1:25 bacteria:cells. **(A)** At each time point, the content of the wells was collected, serially diluted, and plated on TSA for CFU count. **(B)** Light microscopy images of NCI-H441 at 7, 17, and 25 h post-infection. CTRL indicates un-infected NCI-H441 cells at 25 h of culture.

*P. aeruginosa* growth (PA1 strain) in the ALI model was monitored at different time points following infection of differentiated lung epithelial NCI-H441 cells. As shown in Figure 1A for a representative experiment, the bacterium grew well in the model, rapidly increasing the CFU number until 17 h. At 25 h post-infection, the CFU number remained quite stable, but it was associated with an evident cytotoxic effect toward the monolayers as assessed by light microscopy (Figure 1B). Therefore, 7 h was chosen as the best compromise between bacterial growth and *P. aeruginosa*-mediated cytotoxicity as treatment duration with CFS in the subsequent experiments.

### 3.2 Antipseudomonal activity of CFS in the ALI lung infection model

**Figure 2:**
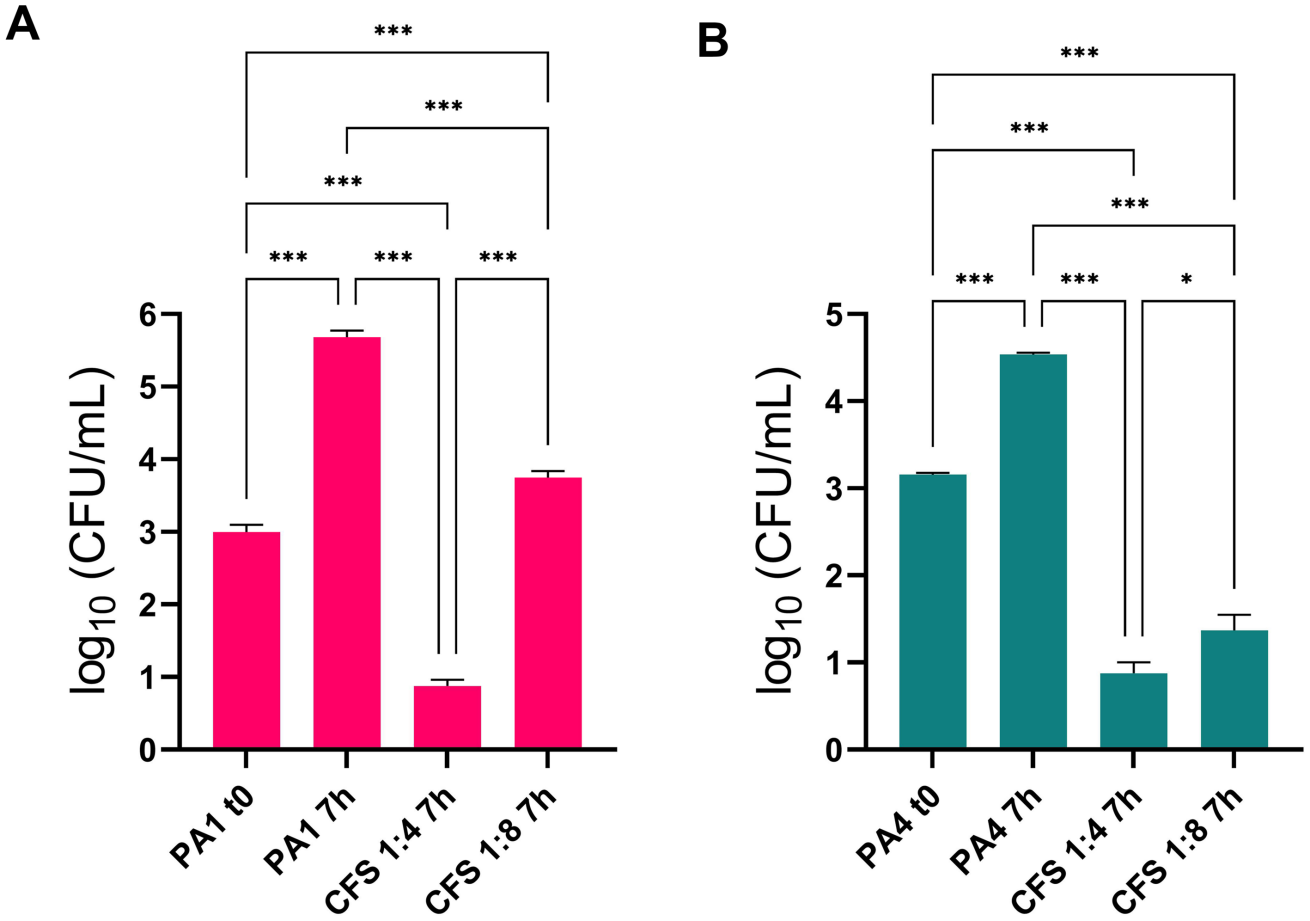
Antipseudomonal activity of CFS in the ALI lung infection model. PA1 and PA4 were used to infect differentiated lung epithelial NCI-H441 cells at a multiplicity of infection of 1:25 bacteria:cells. After 7 h of incubation, CFU numbers in CFS-treated and untreated samples were quantified. **(A)** PA1 non mucoid strain; **(B)** PA4 mucoid strain. * *p* < 0.05, *** *p* < 0.001.

**Figure 3:**
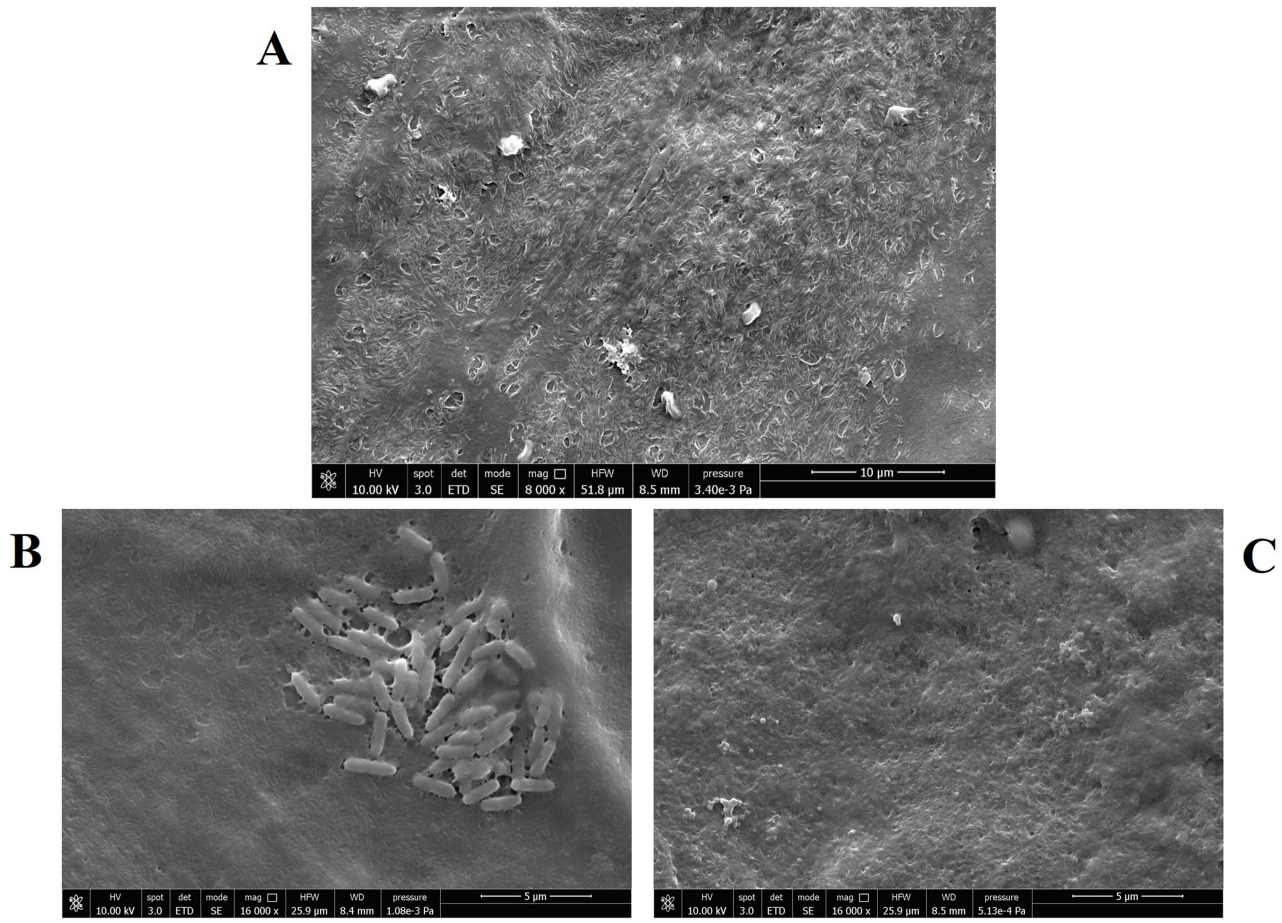
Scanning electron microscopy images of NCI-H441 cells at the ALI. **(A)** Uninfected and untreated monolayers; **(B)** monolayers infected with *P. aeruginosa* strain PA4 (mucoid), untreated; **(C)** monolayers infected with *P. aeruginosa* strain PA4 (mucoid) and treated with CFS 1:8.

Two lung isolates of *P. aeruginosa*, PA1 (non-mucoid) and PA4 (mucoid), were added to differentiated lung epithelial NCI-H441 cells at a MOI of 1:25, and soon after, treated with CFS at 1:4 and 1:8 dilution or left untreated. In untreated samples, both strains showed a marked ability to grow in the ALI model, increasing their viable count in 7 h by approximately 3 Logs (PA1, Figure 2A) and 1.5 Logs (PA4, Figure 2B). Treatment of both strains with CFS at a dilution of 1:8 for 7 h resulted in a significant reduction of the viable count that reached 2 logs for PA1 (Figure 2A) and 3 logs for PA4 (Figure 2B). The 1:4 dilution reduced the CFU number of both strains to the detection limit (10 CFU/mL) (Figures 2A,B).

The marked anti-pseudomonal activity of CFS was confirmed by SEM imaging. Figure 3 A shows uninfected NCI-H441 cells consisting of a continuous layer with morphological features of a polarized epithelium, e.g., the presence of cilia. In samples containing monolayers infected with *P. aeruginosa* and left untreated (Figure 3B), bacterial clusters were clearly visible, resembling the early phase of biofilm formation, while such aggregates were virtually absent in infected and treated samples (Figure 3C).

### 3.3 Cytotoxicity of CFS in the ALI model

**Figure 4:**
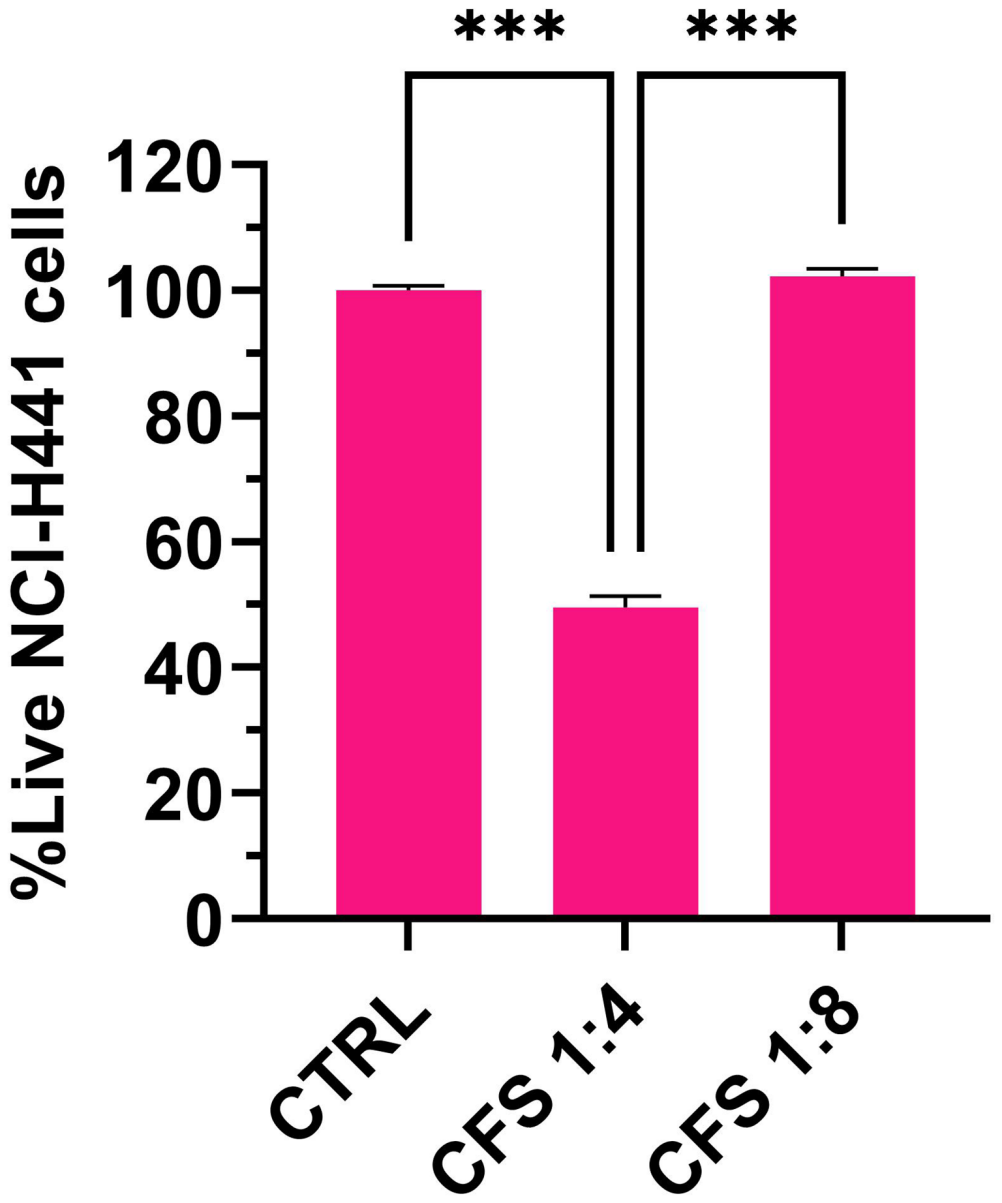
Flow cytometric determination of the cytotoxic effect of CFS on human lung epithelial cells in the ALI model. The figure shows live cell percentages following 7 h exposure to CFS diluted 8 or 4 times in complete RPMI. Control cells were grown in RPMI, 25% MRSB. Mean values ± SEM are shown, *n* = 3. *** *p* < 0.001.

The cytotoxic potential of CFS against differentiated NCI-H441 cells was assessed by flow cytometric determination of propidium iodide uptake. As shown in Figure 4, no cytotoxicity was detected at the active concentration of 1:8 in the ALI model, while CFS diluted 1:4 exhibited a marked cytotoxic effect.

### 3.4 CFS as solution for nebulization

In order to evaluate the possible application of CFS as solution for nebulization, pH and osmolarity were first determined, resulting in a pH of 3.8 and 438 mOsm/Kg, respectively. Subsequently, a preliminary investigation on CFS aerosolization was performed by using an Aerogen® Solo as a vibrating mesh nebulizer. The basic TSI Ph. Eur. method was applied. Lactic acid was used as a tracer for all the experiments, and the deposition profile in the TSI resulted in 19 ± 1% and 76 ± 8% in the first and second stages, respectively. Most of the delivered CFS was thus collected in the second stage, corresponding to particles with an average aerodynamic diameter lower than 6.4 μ m (Hallworth and Westmoreland, 1987), suited to bronchi and deep lung delivery.

**Figure 5:**
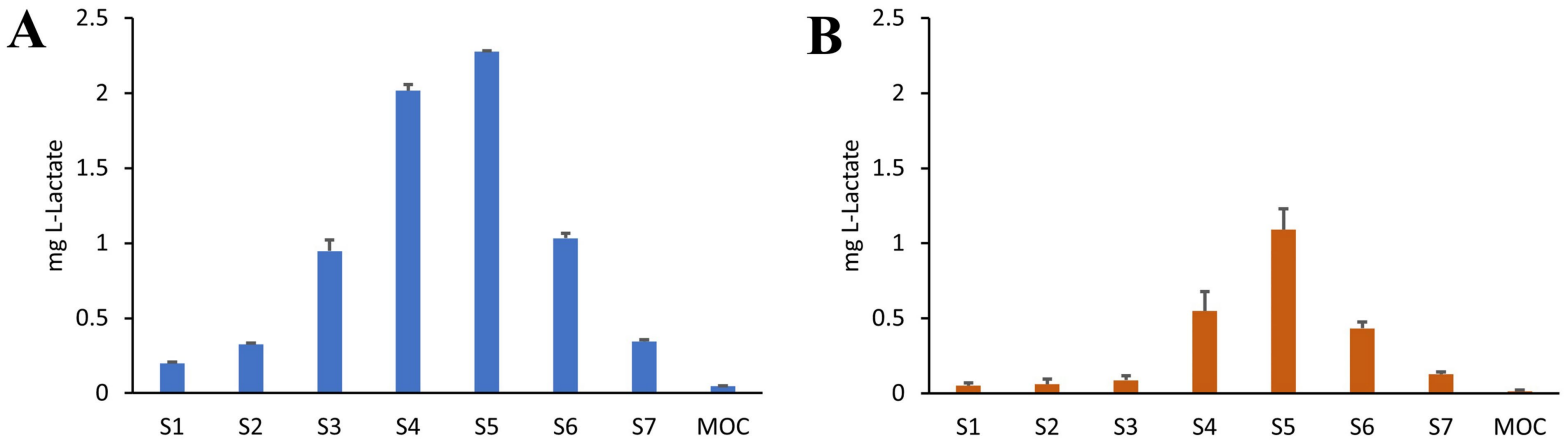
APSD analysis of CFS nebulized with Aerogen® Ultra mesh nebulizer under healthy adult breathing profile **(A)** and adult CF patient profile **(B)**. Lactic acid was used as a tracer for CFS distribution. Amount recovered in Stages (S1–S7) and micro-orifice collector (MOC) are reported (*n* = 5).

APSD was performed by using an NGI coupled to a breathing simulator, applying either a healthy adult profile (as indicated for quality assessment in Ph. Eur.) or a CF patient profile, for an *in vitro*/*in vivo* simulation of aerosol administration. As reported in Figure 5, the distribution of the tracking compound lactic acid across the different stages of the NGI is not significantly affected by the applied breathing profile. CFS is primarily recovered in the final stages that simulate the lower airways, confirming the data from TSI analysis.

For both respiratory profiles, the DDU was determined as well as the fine particle fraction (FPF), defined for particles with an average aerodynamic diameter lower than 5 μm, the mass median aerodynamic diameter (MMAD), and the geometric standard deviation (GSD) describing the uniformity of the distribution. Results are shown in Table 1. Both profiles allowed for a high FPF percentage, MMAD of about 3 mm, and a homogeneous diameter distribution (GSD < 2). However, for CF patients, a lower delivered dose was recorded, corresponding to half of the dose determined for healthy adults. Indeed, this difference can be attributed to the varying I: E ratio between the two profiles.

**Table 1:** APSD of CFS, according to NGI deposition with healthy adult and CF breathing profile.

	Healthy adult profile	Cystic fibrosis profile
Delivered dose (mg)	7.47 ± 0.21	3.17 ± 0.11
FPF < 5 μm (%)	73.1%	68.0%
MMAD (μm)	3.24	2.73
GSD	1.696	1.514
*R* ^2^	0.997	1.0

Delivered dose, Fine particle fraction (FPF < 5 μm), Mass median aerodynamic diameter (MMAD), and Geometric standard deviation (GSD) are shown.

### 3.5 Physicochemical and antibacterial CFS stability under stressed temperature conditions

**Figure 6:**
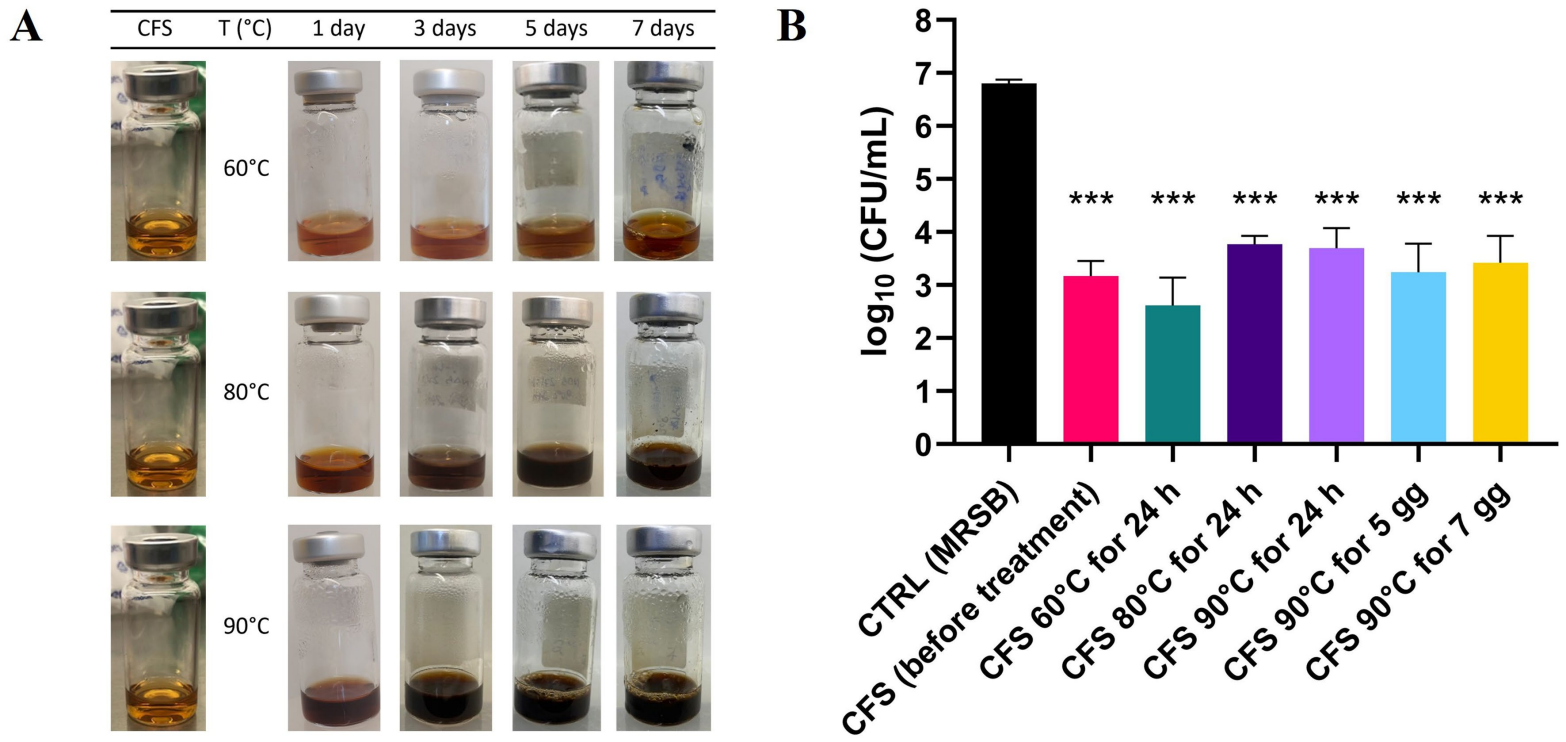
Physicochemical and antibacterial CFS stability under stressed temperature conditions. **(A)** Visual appearance of CFS exposed to high temperatures for different days during the stress stability study, highlighting the browning effect. **(B)** Effect of heat-treatment on antibacterial activity against PA1. Heat-treated CFS were diluted 1:4 in TSB and incubated with PA1 for 45 min at 37°C. Surviving bacteria were quantified by CFU counting on TSA. CTRL represents bacteria treated with MRSB. Data are shown as mean ± SEM from one experiment performed in triplicate. *p < 0.001 *vs*. CTRL (one-way ANOVA followed by Tukey–Kramer multiple comparisons test).

When exposed to high temperatures (up to 90°C), CFS exhibited temperature-dependent darkening (Figure 6A), likely due to Maillard reactions between glucose and protein residues. The darkening reaction followed zero-order kinetics, with an R^2^ value close to 1. Using the Arrhenius plot, once validated, predictions were made regarding the physical stability of the formulation at 25°C, 4°C, and −20°C. Degradation studies were carried out, revealing that maximum browning occurred after 7 days of exposure at 90°C.

Despite these visual changes, all heat-treated CFS samples, whether incubated at 60°C, 80°C, or 90°C for 24 h, or at 90°C for 5 or 7 days, significantly reduced *P. aeruginosa* PA1 viability compared to the control treated with MRSB (Figure 6B). Instead, no statistically significant difference was observed among samples exposed to heat-treated CFS and CFS before treatment, indicating that antibacterial activity was preserved across all tested conditions.

**Figure 7:**
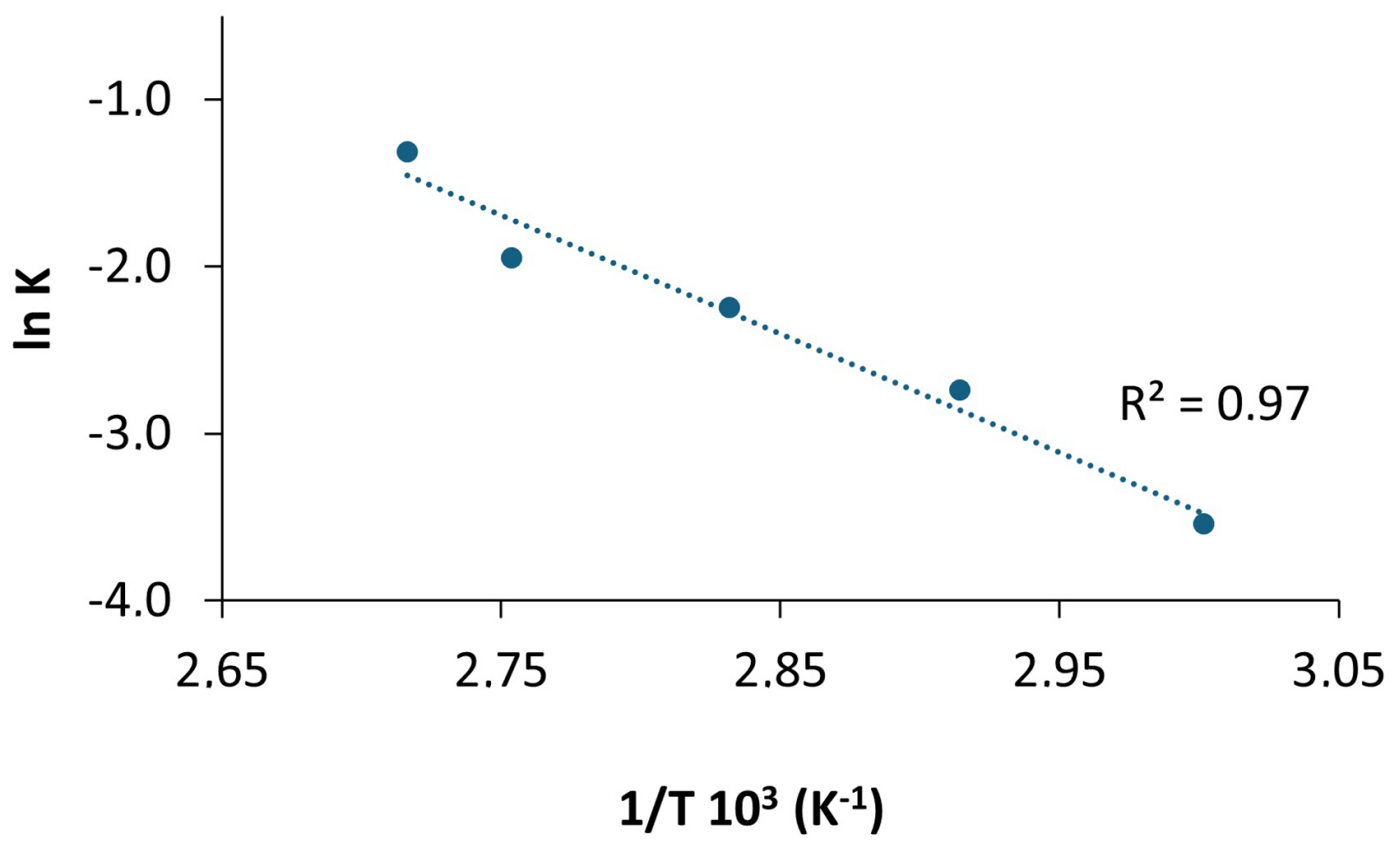
Plot of lnK *vs* 1/T based on the Arrhenius equation. The slope (−Ea//R) and intercept (lnA) were used to calculate reaction parameters. Experimental validation at 70°C and 95°C confirmed consistency with the predicted data.

To construct the Arrhenius plot, the percentage of darkening was assessed, with 100% defined as the darkening observed at 90°C for 7 days, and 0% corresponding to the initial state of CFS. The Arrhenius equation is expressed as lnK = lnA - (Ea/RT), where K represents the reaction rate constant, A is the pre-exponential factor, Ea is the activation energy, R is the gas constant, and T is the absolute temperature. The reaction rate constant (K) was obtained from the slopes of the linear plots, where the darkening percentage was plotted on the y-axis and the number of days on the x-axis. For the Arrhenius plot (Figure 7), lnK was graphed against 1/T, producing a straight line with a slope of -(Ea/R) and a y-intercept of lnA. From this linear regression, values for A and Ea were determined. By substituting the desired temperature into the equation, the reaction rate constant (K) for each temperature was calculated. Model validation was conducted by predicting degradation at 70°C and 95°C, followed by experimental studies at these temperatures. The K values from these degradation studies were comparable to the predictions, and the new data points fit well within the existing Arrhenius plot of lnK versus 1/T. A criterion conventionally recognized in pharmacopeias defines the stability of a formulation as the time between production and the point at which its stability decreases by 10%, signifying the onset of noticeable degradation (European Medicines Agency, 1995, 2003). Based on the evaluation of color change, monitored by UV–Vis absorbance, the Arrhenius plot predicted a prolonged physical stability for CFS at 25°C, with 10% of color variation expected to occur after approximately 9 years of long-term storage. The calculated data for lower temperatures, such as 4°C and −20°C, were even greater.

### 3.6 One-year stability of CFS antipseudomonal activity

**Figure 8:**
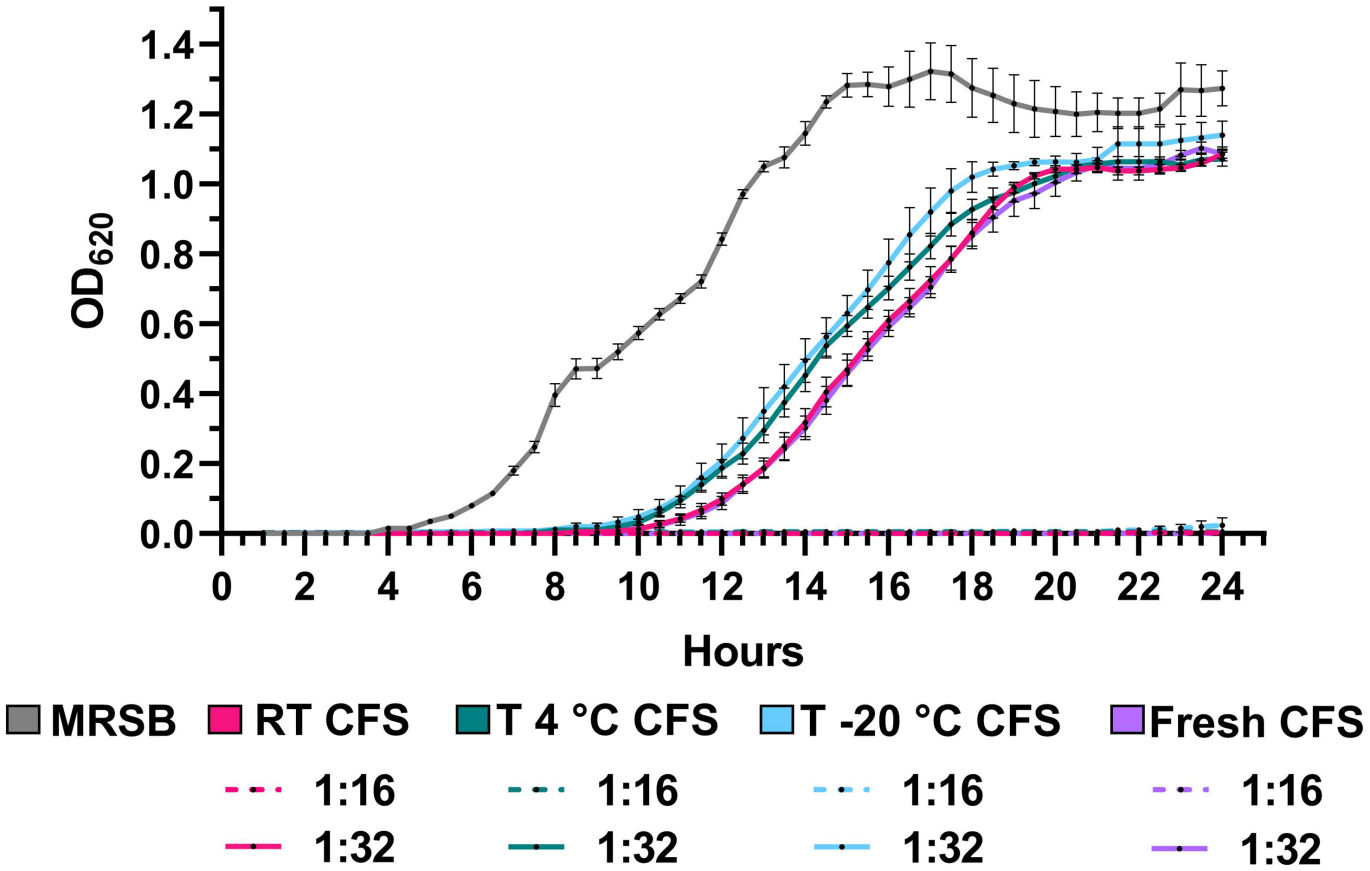
Growth kinetics of PA1 strain in the presence of CFS after 1 year of storage at room temperature (RT), 4°C, and −20°C. Bacteria were incubated with CFS diluted 1:16 and 1:32, and growth was monitored by OD_620_ measurement every 30 min for 24 at 37°C. MRSB indicates bacteria exposed to TSB/MRSB under the same conditions. Data are shown as mean ± SEM from one experiment performed in quadruplicate. (Two-way ANOVA followed by Tukey–Kramer multiple comparisons test).

Antipseudomonal activity of CFS was evaluated following 1 year of storage at room temperature, 4°C and −20°C and compared with that exerted by a freshly prepared batch of CFS. The growth kinetics of *P. aeruginosa* PA1 exposed to CFS stored under the different conditions were almost superimposable between them and with that obtained in the presence of fresh CFS at all the dilutions tested. As shown in Figure 8, the gray line, up to a CFS dilution of 1:16, the growth of *P. aeruginosa* PA1 was completely inhibited, contrary to what was observed with bacteria incubated with MRSB/TSB. At the 1:32 dilution, all CFS samples showed a significant delay in bacterial growth compared to the MRSB control, regardless of storage conditions, with no difference compared to fresh CFS. These results suggest that, despite long-term storage, CFS efficiently retained their antibacterial activity (Figure 8).

## 4 Discussion

There is growing interest in postbiotics as an alternative to live probiotics in clinical applications. This is largely due to their ease of production, safety, extended shelf life, patentability, and lack of risk for antibiotic resistance transfer. Moreover, postbiotics offer additional advantages over live bacteria, such as the possibility of encapsulation for targeted delivery and safe administration to immunocompromised individuals (Kumar et al., 2024).

Despite ongoing challenges in postbiotics development and applications, numerous clinical trials have yielded promising results (for a recent and comprehensive review on the topic, see Kumar et al., 2024). Most of these trials have focused on the digestive tract; however, there is increasing interest in exploring postbiotics for other body sites, including the respiratory system (Mosca et al., 2022; Kumar et al., 2024).

In this study, we investigated the potential of postbiotics from *L. rhamnosus*, in the form of CFS, as an innovative strategy to treat *P. aeruginosa* lung infections. The first step of the work was to test the ability of CFS to reduce *P. aeruginosa* load in an *in vitro* model of lung infection strictly recapitulating the conditions found in human airways. The use of *in vitro* cell culture models is increasingly favored to adhere to the 3R principle (reduce, refine, replace) and limit experimental animal testing for drug screening (Baldassi et al., 2021). Herein, we employed an ALI model, which is considered more physiologically relevant than traditional submerged models (Lacroix et al., 2018). Our group has previously used this model to successfully assess the therapeutic potential of non-conventional antimicrobials (Grassiri et al., 2024). The key feature of ALI culture is that the apical surface of cells is exposed to air while the basal side is in contact with a liquid cell culture medium. This configuration allows cell polarization and differentiation toward a mucociliary phenotype, simulating a scenario close to *in vivo* conditions (Baldassi et al., 2021). The model effectively supported the growth of lung isolates of *P. aeruginosa* (both non-mucoid and mucoid), which caused significant toxicity to the human NCl-H441 distal lung epithelial cell line within 25 h. This observation aligns with the previously reported ability of *P. aeruginosa* to cause lung injury through the secretion of its virulence factors that may be even injected directly into the target cells via a specialized TTSS (type III secretion system) widely expressed in environmental as well as clinical isolates (Ader et al., 2005). Nevertheless, CFS markedly reduced (2–3 Log) the *P. aeruginosa* viable count in approximately 7 h, a time point at which the epithelial layer remained intact. In contrast, untreated samples showed clear signs of bacterial aggregation within the same time frame, resembling early biofilm formation as confirmed by SEM imaging. This finding is consistent with the rapid biofilm-forming ability observed in certain clinical *P. aeruginosa* isolates, which can initiate biofilm development in as little as 2 h *in vitro* (Olivares et al., 2016). This suggests that similar dynamics may occur *in vivo*, under conditions comparable to those replicated in this study. SEM imaging of untreated samples also showed the formation of a sort of “bridges” among bacterial cells that may indicate the early production of extracellular polysaccharides (e.g., Psl) previously demonstrated to hold bacteria together at the early stage of biofilm development on a surface (Ma et al., 2009). The rapid mechanisms of action demonstrated in the ALI infection model could therefore represent an important property of CFS, capable of preventing the initial stages of biofilm formation before the acquisition of a highly tolerant phenotype by the bacterium. The rapid mechanism of action of CFS corroborates findings from a previous study conducted in a cell-free system, which demonstrated the absence of bacterial regrowth even at extended time points (up to 24 h) (Pompilio et al., 2024).

In a recent study, we sought to identify the components of the CFS responsible for their antipseudomonal activity by fractionating the CFS and analyzing their constituents using a proteomic approach (Bianchi et al., 2025). Our findings indicate that lactic acid—present at approximately 125 mM in the undiluted CFS—plays a significant role in their antibacterial effect. However, additional components (proteins) are likely to contribute to the overall antimicrobial activity (Bianchi et al., 2025).

Importantly, the anti-pseudomonal activity of CFS in the ALI infection model occurred at non-toxic concentrations (dilution 1:8) for lung epithelial cells as assessed in cytotoxicity assays. However, the therapeutic window was quite narrow, as at one dilution lower (1:4), a detectable toxicity was observed. It is worth noting that *in vitro* toxicity assays may not accurately replicate the complex interactions established in a living organism where cells lie on the basal membrane, are protected within a tissue and are surrounded by the host extracellular matrix. Supporting this, our recent in vivo study using the *Galleria mellonella* model showed no cytotoxic effects from *L. rhamnosus* GG CFS, which also conferred protection against subsequent *P. aeruginosa* infection (Pompilio et al., 2024). Additionally, previous research has reported higher cytotoxic potential of CFS from lactobacilli toward immortalized cell lines than primary cells (Motevaseli et al., 2013). Despite these considerations, the possible toxic effects of CFS should be further investigated and, eventually, strategies aimed at minimizing them should be implemented. For instance, we previously demonstrated that encapsulation of a relatively toxic antimicrobial peptide in chitosan nanoparticles led to a reduction of its toxic potential against murine cells while preserving its antibacterial potential that was rather reinforced by the presence of chitosan (Piras et al., 2015). Similarly, Sharaf et al. (2019) applied this approach to produce seven lactic acid culture-free supernatants in nano-chitosan-loaded forms. In another recent study, CFS of *Bifidobacterium bifidum* and *L. acidophilus* were successfully encapsulated in chitosan nanoparticles by the ionic gelation technique (Derakhshan-Sefidi et al., 2024) for oral administration. Encapsulation enhanced their antibacterial activity against *Vibrio cholerae*, improved their stability during simulated gastric transit and enabled their controlled release at the site of interaction between the host and bacteria (Derakhshan-Sefidi et al., 2024). Thus, although still relatively under-investigated, CFS encapsulation strategies may contribute to accelerating the clinical application of CFS by reducing their toxicity and enhancing their activity.

The next phase of our study focused on evaluating the delivery of CFS to the lungs as part of their preclinical assessment. The International Scientific Association of Probiotics and Prebiotics (ISAPP) defines postbiotic, “a preparation of inanimate microorganisms and/or their components that confers a health benefit on the host” (Salminen et al., 2021). Historically, however, the term postbiotics has often referred to secreted metabolites. Despite postbiotics that act purely through physical mechanisms could be classified as medical devices (European Parliament, 2017), many postbiotics, especially bacterial lysates, have been used for medical purposes, in vaginal, nasal and oral routes of administration (Vinderola et al., 2023). Among postbiotics, bacterial lysate-based medicines are widely used for the treatment or prevention of upper and lower respiratory tract infections, as well as chronic respiratory disease. These medicines are mostly administered orally (capsules, tablets, granules/powder to form a mixture, or oral drops), or inhaled through the nose (as a liquid) (European Medicines Agency, 2018). Moreover, biological medicinal products, as defined in Annex 1 of Directive 2001/83/CE, are those whose active substances are biological in origin. Since postbiotics are derived from living organisms, they can be considered in the biological medicinal products category and, as such, require physical–chemical-biological testing as part of stability and quality programs. Although specific guidelines for postbiotics are lacking, developers can follow existing protocols for biological medicinal products (Vinderola et al., 2023).

In this contest, the translational potential of CFS as a solution for nebulization, was evaluated in accordance with Eu. Phar. (European Pharmacopoeia, 2023) indications and EMA guidelines (European Medicines Agency, 2024). Firstly, sterility was guaranteed by the last step of the preparation method, when CFS are filtered through 0.22 μm filters. The solution’s pH (3.8) fell within the Eu. Phar. range of pH 3–8.5, and its osmolarity was indicative of a slightly hypertonic solution (438 mOsm/Kg), within the tolerability range for inhalable formulations (150–550 mOsmol/kg) (Law, 2001).

Mesh nebulization technology was chosen because it generally does not compromise the stability of labile products and has recently demonstrated superior performance when tested for the delivery of *Lacticaseibacillus rhamnosus GG* suspension (Byun et al., 2023). A preliminary evaluation confirmed the CFS as suitable for aerosol administration, leading to almost 80% of the solution recovered in the second stage of a TSI. Given that the epithelial lung lining fluid (ELF) physiologically dilutes inhaled droplets, the use of undiluted CFS for nebulization in this study was chosen to reflect clinically relevant exposure conditions. A 2 mL dosage was applied as a representative volume, allowing for both realistic delivery simulation and accurate analytical quantification during the aerodynamic particle size distribution (APSD) studies. This strategy will inform the design of future studies aimed at determining the appropriate clinical dosage, based on the effective and cytocompatible 1:8 dilution. The setting of the therapeutic protocol will take into account that the ELF volume is estimated to range from 10 to 70 mL, with distribution varying by lung region and disease state; specifically, in the respiratory region—where CFS is intended to act—it is estimated at 7–20 mL (Hastedt et al., 2016; Hastedt et al., 2022). In view of future applications, additional investigations were performed to ensure CFS’s effective delivery to the deep lungs. Particularly, APSD is considered one of the Critical Quality Attributes (CQA) of inhalation products, crucial to ensure consistency for further *in vivo* studies. Instead of using the percentage of emitted dose for the TSI analysis, a multistage impactor or impinger should be used to measure the distribution on each stage and cumulative undersize mass, as these provide more accurate dose variations. A cumulative percentage versus cut-off diameter plot should also be included, from which MMAD and GSD can be determined (European Medicines Agency, 2024). For the APSD measurements performed, mass balance reconciliation was always in the 98–85%, confirming the reproducibility of the analysis. Furthermore, it is recognized that depending on the disease and disease severity, patient-breathing capability and in particular, the flow rate can make a difference in the inhaled and fine particle doses. Lastly, it affects the impact of the drug on lung regions. To assess the complete delivery profile of the medicinal product for *in vivo* studies (pivotal clinical and/or comparative), *in vitro* characterization for both the delivery rate and total amount delivered should be provided. A recognized validated technology to ensure accurate *in vitro/in vivo* correlation is a breath simulator coupled to NGI (European Medicines Agency, 2024). In this study, the aerosol was generated using the same nebulizer and settings as those supposed for future use in adult CF patients. The performed analysis evidenced the good aerodynamic distribution of the CFS both under simulated healthy adult and adult CF patients’ breathing profiles. The most affected parameter is the delivered dose, which is drastically reduced for the simulated CF patient’s profile and suggests a dose volume adjustment to provide a comparable effective dose impact.

In the case of inhalation therapy development, animal models are necessary and widely used for preclinical safety and pharmacological testing of drugs. Nevertheless, fundamental anatomical differences exist between the airways of rodents and humans, emphasizing a substantial lack of functional homology regarding drug deposition rates and localization (Baldassi et al., 2021). For instance, mice only have 6–8 levels of branching airways while humans have up to 20 or more. Furthermore, mice do not have respiratory bronchioles comparable to humans, which are characterized by interruptions on their walls that project into the alveoli. They only have short terminal bronchioles opening straight into the alveolar ductules (Bal and Ghoshal, 1988). Therefore, while data derived from rodent models cannot be directly translated to human contexts, the use of a breathing simulator coupled with NGI is essential to confirm the feasibility of human administration and assess dose impact. In this study, the combination of *in vitro* cellular models and *in vitro* ASPD under simulated breathing conditions has advanced our understanding, demonstrating that the product is effective and suitable for inhalation delivery. The necessary animal testing will follow to confirm the pharmacological and safety aspects.

In view of the future development of CFS as a solution for inhalation, stability issues were also investigated. A key factor driving interest in postbiotics is their natural stability, both during production and storage. Live microorganisms present a technological challenge because they are often sensitive to oxygen and heat. However, postbiotics, being non-living, can be easily developed into products with a long shelf life. Postbiotics may also be better suited for regions that lack reliable cold chains or face challenges with ambient temperatures that hinder the storage of live microorganisms (Salminen et al., 2021). For example, the marketed product Lantigen B (Bruschettini S.r.l.), an oral suspension of bacterial lysate, has an expiration date of 3 years in unopened packaging, under storage at temperatures not exceeding 25°C. Several literature studies related to postbiotics are actually focused on isolated metabolites, antimicrobial peptides, and bacteriocins, which actually do not fall within the ISAPP definition of postbiotics. These molecules have a fair stability at diverse pH, UV exposure, and temperatures (Hasan et al., 2025), e.g., confirming a 30-day stability at room temperature (Janakiraman, 2012). CFS may offer even greater stability, with several studies reporting 1 to 5 months of stability at temperatures ranging from 4 to 35°C (Koohestani et al., 2018; Mani-López et al., 2022). Thanks to the interest in CFS application in food processing, it was assessed that when the antimicrobial activity is mainly due to organic acids, phenyllactic acid, fatty acids and heat-resistant proteinaceous compounds, the CFS supports baking (up to 14 min at 210°C) and heat sterilization (Cortés-Zavaleta et al., 2014; Schmidt et al., 2018).

In this study, physicochemical stability studies of CFS were undertaken under stress temperature conditions. Specifically, the Arrhenius equation was applied after exposing CFS sealed into glass vials to extreme temperatures for short periods. The goal was to identify the primary degradation kinetics and extrapolate its behavior under the conditions outlined in the International Council for Harmonization of Technical Requirements for Pharmaceuticals for Human Use (ICH) guidelines (European Medicines Agency, 1995, 2003). These studies are crucial not only for assessing the stability of CFS but also for understanding how it may respond to prolonged storage and heat stress. In addition to functionality tests, the evaluation included acceptance criteria related to appearance and physical attributes—such as color changes and phase separation—which must be monitored and reported for the final drug product (European Medicines Agency, 1995, 2003).

A main macroscopical change observed in heat-stressed CFS was a temperature and time-dependent darkening. This phenomenon is attributed to Maillard reactions between glucose and protein residues, which can also lead to the formation of a visible particulate when the highest darkening is reached (Jiang et al., 2019). This effect was monitored by instrumental acquisition of UV–Vis absorbance. The results evidenced that 10% color alteration would be expected at 9 years of storage at 25°C, thus predicting a good stability performance of CFS.

Biological activity is a fundamental criterion in stability assessment. Firstly, it was observed that no statistically significant differences were observed between heat-treated and untreated CFS in their antibacterial activities, despite the heat-induced degradation. These findings agree with previous reports in which the antibacterial activity of CFS was retained after heating at 95°C for 10 min (Pompilio et al., 2024). It is noticeable that the forced degradation induced the formation of Maillard reaction products (MRPs), which are widely investigated as antimicrobial agents and could then participate in the antimicrobial effect for the heat-stressed CFS (Habinshuti et al., 2019; Nooshkam et al., 2020). To complete the stability studies, the antipseudomonal activity of CFS at one-year storage at room temperature, at 4°C and at −20°C was assessed. The result obtained demonstrated that the killing ability of CFS against *P. aeruginosa* remained unaffected under all three long-term storage conditions, thereby providing additional evidence in support of their pre-clinical characterization.

In conclusion, in an ALI lung infection model, CFS from *L. rhamnosus* demonstrated effective anti-bacterial activity against both non-mucoid and mucoid strains of *P. aeruginosa* isolated from CF lung. The liquid formulation developed demonstrated favorable physicochemical characteristics for pulmonary administration, with optimal aerodynamic particle size distribution for deep lung penetration, essential for addressing chronic infections. Furthermore, CSF exhibited a marked long-term stability, along with the lack of cytotoxicity at microbicidal concentrations. These findings support the development of postbiotic-based inhalation therapies in the context of rising antimicrobial resistance.

## Data Availability

The raw data supporting the conclusions of this article will be made available by the authors, without undue reservation.
